# The financial burden of an influenza outbreak in a small rehabilitation centre

**DOI:** 10.1111/irv.12696

**Published:** 2019-10-25

**Authors:** Parham Sendi, Sarah Dräger, Bettina Batzer, Susanne Walser, Marc Dangel, Andreas F. Widmer

**Affiliations:** ^1^ Division of Infectious Diseases and Hospital Epidemiology University Hospital Basel University of Basel Basel Switzerland; ^2^ Institute for Infectious Diseases University of Bern Bern Switzerland; ^3^ Bürgerspital Basel Reha Chrischona Bettingen Switzerland

**Keywords:** cost‐of‐illness, disease burden, economic burden, influenza, influenza outbreak, productivity

## Abstract

We report an influenza outbreak in a 75‐bed rehabilitation centre and present the detailed microeconomic impact that it had during the season 2016/2017. The direct medical, direct non‐medical and indirect costs were calculated. The outbreak included 18 patients with influenza and 8 contact patients, leading to 86 days with isolation precautions. During the outbreak month, 25 (15%) employees were absent from work for 89 days (mean 3.6 days, SD ± 1.8), and during the entire influenza season 33 for 175 (5.3 ± SD 4.6) days, respectively. The economic burden related to the outbreak was 114 373 CHF (106 890 €, 112 131 $).

## 1 INTRODUCTION

Seasonal influenza results in considerable morbidity and mortality, as well as increased healthcare utilization and costs.^1^ Each year, the disease leads to substantial losses in productivity for both patients and caregivers.^2^ The economic impact of influenza is of interest at both the microeconomic and the macroeconomic level.^3^ Although several studies have calculated the cost‐effectiveness of vaccination programs^4^ or applied modelling tools,^5^ precise data on the direct and indirect costs of influenza for a single institution are scarce. Here, we report an influenza outbreak in a rehabilitation centre and present its detailed microeconomic impacts.

## 2 METHODS

### 2.1 The outbreak

The institution Reha Chrischona (Bettingen, Switzerland) consists of 75 beds (one‐bed or two‐bed room[s]) and provides between 19 000 and 20 000 days of nursing care each year.^6^ Specialized in the field of internal medicine, musculoskeletal rehabilitation and oncology rehabilitation, the institution employed 169 individuals in 2017. Standard precautions are applied to the care of all patients. Routine influenza vaccination is offered to healthcare workers without a mandatory vaccine policy. Influenza A subtype H3N2 was the predominantly circulating virus in the influenza season 2016/2017, the time interval defined to be from December 2016 to February 2017.^7^ The outbreak in the institution occurred in January 2017.

### 2.2 Outbreak management

Influenza outbreak management was implemented as described elsewhere,^8^ after the detection of one confirmed influenza case and four patients with an influenza‐like illness (ILI). An ILI case definition was implemented and included acute onset of new symptoms (ie, within 24 hours). The presence of fever (≥38°C) and cough, or fever and other respiratory illness symptoms or fever without a reasonable alternative diagnosis was required to fulfil the case definition.^9^ A rapid influenza diagnostic test (polymerase chain reaction [PCR], Xpert Flu, Cepheid) was performed in nasopharyngeal swab specimens obtained from ILI cases. The influenza test result was communicated to both infection control specialists and healthcare personal of the institution within 24 hours. Daily active surveillance for acute respiratory illness among all patients and healthcare personnel was started and continued for 18 days after the last laboratory‐confirmed influenza case was identified. Combined droplet‐contact isolation precautions were applied to all patients who fulfilled ILI case definitions. The importance of standard precautions was stressed daily, and all healthcare personnel and visitors were requested to wear surgical masks and to perform hand hygiene before and after touching patients (irrespective of whether or not they had contact with an ILI case). Patients who fulfilled ILI case definitions remained in their private room or were discharged at home, depending on their health status and completion of rehabilitation programme. If patient transport to another institution was necessary, patients had to wear surgical masks during the transport and information on the influenza test result was communicated. Isolation precautions in confirmed influenza cases were applied for at least five days after illness onset (at least ten days in immunocompromised patients) or until 24 hours after the resolution of fever and respiratory symptoms, whichever was longer. Oseltamivir (75 mg) was administered twice daily to all patients who fulfilled the ILI case definition and was continued in laboratory‐confirmed cases for five days or in immunocompromised patients for ten days. PCR was repeated in immunocompromised patients with confirmed influenza after five or ten days of antiviral treatment. Patients exposed to a PCR‐positive influenza patient in the same patient room were pre‐emptively treated with oral oseltamivir (75 mg twice daily) for five days. Antiviral chemoprophylaxis was only offered to the defined patient group but not to all individuals.

### 2.3 The economic burden

Data (direct costs and number of absentees from work per day during the outbreak) for the calculation of the economic burden were collected prospectively for this study. To calculate the economic burden, we estimated the direct medical costs, direct non‐medical costs and indirect costs.^3^ We defined direct medical costs as those associated with diagnostics and treatment of patients with ILI and their room neighbours (ie, contact patients). Direct non‐medical costs included the extra expense for droplet‐contact isolation precautions. These costs were calculated in a previous investigation and extrapolated for this study.^10^ They included costs for extra materials used (eg, surgical masks, gloves, gowns), increased workload and off isolation activities specifically for patients under isolation precautions.^10^ Indirect costs were defined as the value of lost production because of reduced working time for caregivers (productivity losses). We focussed only on the personnel in the institution because the study targeted on the economic burden of the rehabilitation centre. The institution provided anonymized data for the numbers of employees and the number of work‐absence days during the outbreak month (January 2017). At the end of influenza season 2016/2017, an anonymized questionnaire was distributed to all employees to evaluate their vaccine status, whether they had been exposed to patients with influenza, whether they had experienced an ILI and the number of absence days from work because of ILI. The completed questionnaires were collected by the investigators. Neither occupational health nor human resources of the rehabilitation centre had access to the questionnaires. The productivity loss was calculated as [days of absence] × [median salary for a specific profession within this institution]. The sum of productivity loss was calculated and provided by the chief executive officer of the institution from the results generated by the questionnaire (Table 1). The currency of the productivity loss is Swiss Francs (CHF). The equivalent amount was calculated in Euros (1€ = 1.07 CHF) and US dollars (1$ = 1.02 CHF) on the basis of currency indices in January 2017. Ethics committee approval was not required for this type of study.

**Table 1:** Self‐reported work loss because of ILI during influenza season 2016/2017 at a rehabilitation centre

Profession/Occupation	No. of employees with ILI	No. of work days absent	Mean ± SD
Nursing care	20	99	4.9 ± 4.1
Hotel sector, kitchen, laundry	4	28	7.0 ± 5.1
Physiotherapy, occupational therapy	1	10	–
Social service employees	1	8	–
Others^a^	7	30	4.3 ± 2.4
Total	33	175	5.3 ± 4.5

Abbreviation: ILI, influenza‐like illness.

a Others included cleaning service, reception and administration, patient support, volunteers.

## 3 RESULTS

**Figure 1:**
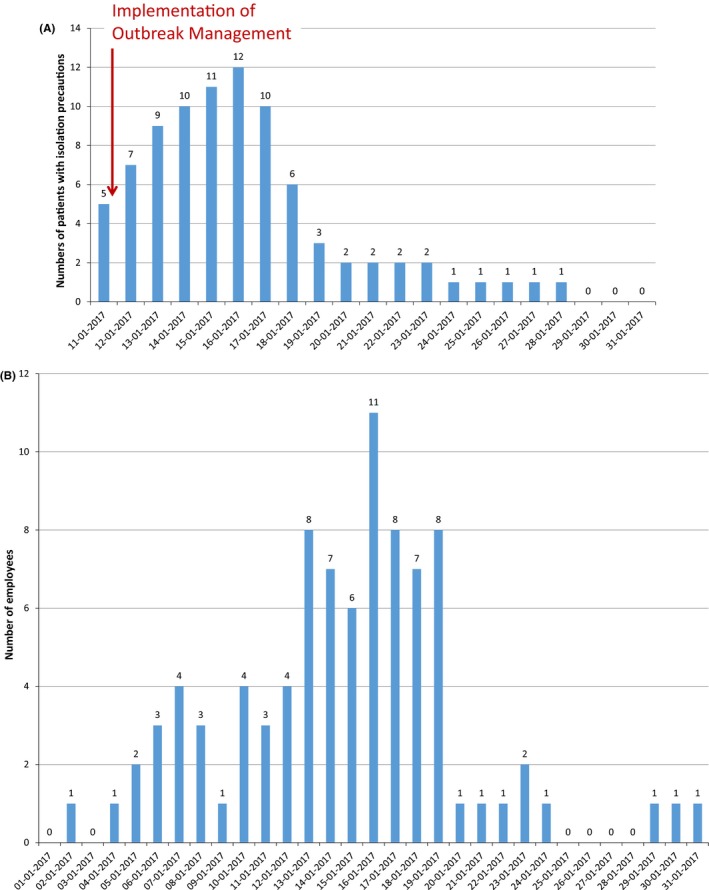
A, The number of patients with contact‐droplet isolation precautions per calendar day during the outbreak. B, Number of absentees per day during the outbreak month. The caregiver absent at January 2nd was not included in the study, because absence was more than 7 days prior to the start of the outbreak

The number of patients with contact‐droplet isolation precautions per calendar day during the outbreak month is summarized in Figure 1A. Eighteen patients had laboratory‐confirmed influenza, and eight contact patients were identified. Two patients with laboratory‐confirmed influenza were referred to a tertiary care centre because of decompensated heart failure, after one day and five days with isolation precautions, respectively. Five patients with laboratory‐confirmed influenza and one patient with ILI and negative influenza test result were discharged at home, because they were in good health and their rehabilitation programme was completed. Their periods with isolation precautions ranged from one day to five days prior to discharge. In total, 86 isolation precautions days were implemented in 18 days. An index case for the nosocomial outbreak was not identified. None of the patients died. No new ILI case was observed after 7 days. The numbers of absentees per day during the outbreak month are shown in Figure 1B. In January 2017, the employer reported that 25 (15%) employees were absent for 89 days (mean 3.6 days, SD ± 1.8, range 1‐8 days per employee). The day with the highest numbers of patients with isolation precautions (16 January 2017; 12 individuals, Figure 1A) coincided with the day of the highest number of absentees from work (11 individuals, Figure 1B).

Of the distributed questionnaires (169), 158 were returned and 150 (89%) were completed and included in the study. Hundred twenty (80%) of 150 employees were caregivers with patient contact, and 90 (60%) were exposed to patients with influenza. Twenty‐three (15%) of 150 responders reported having been vaccinated against seasonal influenza.

Of the 40 (of 150, 27%) employees who reported having experienced ILI during the season, three went for PCR testing and had laboratory‐confirmed influenza. Thirty‐three (22%) of 150 employees reported absence from work for 175 days because of ILI. Twenty‐five (62.5%) of 40 employees with ILI were absent during the outbreak month, and 8 (33.3%) were absent in the other months of the influenza season. Seven employees with ILI reported no absence; their disease period did not coincided with the working days. The mean self‐reported work loss because of ILI was 5.3 ± SD 4.6 (range 1‐21) days. Seven (18%) of these 40 employees were vaccinated against seasonal influenza. The distribution of the number of employees and the days absent from work per profession is shown in Table 1.

The direct costs related to the influenza outbreak are illustrated in Table 2. The sum of the calculated median loss of productivity per profession for 40 employees was 87 000 CHF (81 308 €, 82 294 $). The total economic burden related to the influenza season 2016/2017 was 114 373 CHF (106 890 €, 112 131 $).

**Table 2:** Economic burden of the influenza outbreak and loss of productivity

Currency	Cost per item	Number of items	Total costs		
			CHF	€	$
Direct medical costs					
Medication					
Oseltamivir for ILI/influenza cases^a^	7.50	270	2025		
Antiviral chemoprophylaxis^a^	7.50	80	600		
Diagnostics					
PCR for influenza A/B^b^	204	37	7548		
Total direct medical costs			10 173	9507	9974
Direct non‐medical costs					
Costs for contact and droplet isolation per bed‐day^c^	200	86	17 200	16 075	16 863
Total direct costs			**27 373**	**25 582**	**26 837**
Loss of productivity^d^	33	175	87 000	81 308	85 294
Total economic burden			**114 373**	**106 890**	**112 131**

Note

The equivalent amount of CHF was calculated in Euros (1€ = 1.07 CHF) and US dollars (1$ = 1.02 CHF) on the basis of currency indices in January 2017.

Aabbreviations: ILI, influenza‐like illness; PCR, polymerase chain reaction.

a Variable included oseltamivir for empiric treatment in ILI cases and for targeted treatment in influenza‐confirmed cases. Price per capsule in 2017.

b Costs per item according to reference^14^ in 2017.

c Costs per item were calculated in a previous investigation and extrapolated for this study.^10^

d Calculated as [days of absence] × [median salary for a specific profession within this institution]. The sum of productivity loss was calculated on the basis of data provided in Table 1.

## 4 DISCUSSION

The rapid infection control intervention was effective for the described influenza outbreak. Only 18 patients with laboratory‐confirmed influenza and eight contact patients were involved, and the outbreak period was short (18 days). It generated 27 373 CHF (25 582 €, 26 837 $) in direct costs to the institution. Influenza outbreak management may differ in various aspects from institution to institution, and these differences influence direct costs. The Centers for Disease Control and Prevention (CDC) recommends to consider the use of antiviral chemoprophylaxis for all residents of long‐term care and post‐acute care facilities and to healthcare personnel.^8^ This approach may increase the direct costs for medication and the potential risk of resistance development as well as unwarranted drug effects and interactions with other compounds. The counter argument includes reduction of transmission, because failure to control the outbreak will result in more nosocomial influenza cases and increased costs. The rehabilitation centre consisted of rooms only with one or two beds. Therefore, we focussed on rigorous hygiene precautions for patients, healthcare personal and visitors, and administered antiviral prophylaxis with a targeted approach. It is possible that with a more generous use of antiviral prophylaxis, the infection control could have been even more efficient. However, we observed an excellent compliance for hygiene precautions among the healthcare personal and visitors. No new incidence case was detected one week after its implementation, and none of the patients required isolation precautions 16 days after implementation.

The productivity loss because of individuals with ILI is frequently reported on a macroeconomic level. Molinari et al^2^ estimated the productivity costs for influenza cases in the United States in 2003 and projected lost earnings due to illness to be $16.3 billion (95% confidence interval, $8.7 to $31.0) annually. Putri et al^11^ recently published their results for 2015 and estimated indirect costs at $8.0 billion and ‘days of productivity lost' at $20.1 million. We prospectively focused on the microeconomic cost level at a single institution. We used data from the employer and self‐reported data from the employees. The impact of influenza on work‐related absenteeism can be reviewed from different perspectives in studies involving laboratory‐confirmed influenza, physician‐diagnosed ILI or self‐reported ILI.^12^ The reliability of self‐diagnosis may be lower than diagnosis by a physician or confirmation by laboratory tests. However, all employees in our study were aware of the case definitions for ILI and the vast majority of absentees were caregivers and educated about influenza. The mean reported work loss in our survey (5.3 ± SD 4.6 days per employee with ILI) was in the same range as in previous studies.^12^ Differences in the circulating influenza strains and the proportion of vaccinated individuals may influence the range of absence days because of ILI. Vaccine effectiveness against seasonal H3N2 in working‐age adults was reported to be 35%.^13^ We are unable to evaluate whether there was a shorter duration of work absence for vaccinated patients who contracted influenza than for unvaccinated employees. Nonetheless, the proportion of vaccinated employees was low (15%). Policy change and constant education for healthcare personal may potentially increase this proportion.

The study has limitations. We did not encounter the numbers of surgical masks and the volume disinfectant for hand hygiene used by visitors and personnel without patient contact. Thus, the direct non‐medical costs are likely underestimated. The productivity loss data should be interpreted with caution, because there may be a recall bias of self‐reported absence and the anonymized questionnaire was not previously validated. Also, these data need to be analysed from a seasonal perspective over several years. Nonetheless, in this investigation, we demonstrated the financial damage at a single institution caused by an outbreak. In light of the cost pressure to which rehabilitation centres are exposed, these data may have an implicit effect on influenza prevention strategies. These include programmes to increase the vaccination rates among healthcare personnel and protocols for antiviral chemoprophylaxis. A detailed and rapid outbreak management concept against influenza may turn to be cost‐effective, in particular for small institutions with limited resources.

## CONFLICT OF INTEREST

None.
